# The clinical development candidate CCT245737 is an orally active CHK1 inhibitor with preclinical activity in RAS mutant NSCLC and Eμ-MYC driven B-cell lymphoma

**DOI:** 10.18632/oncotarget.4919

**Published:** 2015-07-22

**Authors:** Mike I. Walton, Paul D. Eve, Angela Hayes, Alan T. Henley, Melanie R. Valenti, Alexis K. De Haven Brandon, Gary Box, Kathy J. Boxall, Matthew Tall, Karen Swales, Thomas P. Matthews, Tatiana McHardy, Michael Lainchbury, James Osborne, Jill E. Hunter, Neil D. Perkins, G. Wynne Aherne, John C. Reader, Florence I. Raynaud, Suzanne A. Eccles, Ian Collins, Michelle D. Garrett

**Affiliations:** ^1^ Cancer Research UK Cancer Therapeutics Unit, Division of Cancer Therapeutics, The Institute of Cancer Research, London, UK; ^2^ Institute for Cell and Molecular Biosciences, Medical School, Newcastle University, Newcastle Upon Tyne, UK; ^3^ Sareum Ltd, Cambridge, UK; ^4^ School of Biosciences, University of Kent, Canterbury, Kent, UK

**Keywords:** CHK1, CCT245737, pharmacology, antitumor activity, biomarker assay

## Abstract

CCT245737 is the first orally active, clinical development candidate CHK1 inhibitor to be described. The IC_50_ was 1.4nM against CHK1 enzyme and it exhibited>1,000-fold selectivity against CHK2 and CDK1. CCT245737 potently inhibited cellular CHK1 activity (IC_50_ 30-220nM) and enhanced gemcitabine and SN38 cytotoxicity in multiple human tumor cell lines and human tumor xenograft models. Mouse oral bioavailability was complete (100%) with extensive tumor exposure. Genotoxic-induced CHK1 activity (pS296 CHK1) and cell cycle arrest (pY15 CDK1) were inhibited both *in vitro* and in human tumor xenografts by CCT245737, causing increased DNA damage and apoptosis. Uniquely, we show CCT245737 enhanced gemcitabine antitumor activity to a greater degree than for higher doses of either agent alone, without increasing toxicity, indicating a true therapeutic advantage for this combination. Furthermore, development of a novel ELISA assay for pS296 CHK1 autophosphorylation, allowed the quantitative measurement of target inhibition in a RAS mutant human tumor xenograft of NSCLC at efficacious doses of CCT245737. Finally, CCT245737 also showed significant single-agent activity against a MYC-driven mouse model of B-cell lymphoma. In conclusion, CCT245737 is a new CHK1 inhibitor clinical development candidate scheduled for a first in man Phase I clinical trial, that will use the novel pS296 CHK1 ELISA to monitor target inhibition.

## INTRODUCTION

The DNA damage response (DDR) pathway has evolved to protect cells from genetic insults in an effort to preserve genomic integrity and cell viability [1, 2]. Anticancer chemotherapy often includes DNA damaging agents which activate the DDR [3]. An important gene in this process is *TP53* which can induce G1/S cell cycle arrest and either DNA repair or apoptosis [4]. A hallmark of many tumors is the lack of functional p53 protein with a consequent loss of the G1/S checkpoint leading to a potential increase in reliance on the S and G2/M checkpoints for survival following genotoxic stress [5]. This has therefore stimulated the development of selective G2 checkpoint inhibitors for combination with DNA damaging anticancer drugs [6-9].

One potential drug target controlling this checkpoint is the serine/threonine kinase CHK1 which has been shown to be involved in the G1 and G2 checkpoints through altering CDC25A stability and CDC25C localization, respectively [3, 10, 11]. CHK1 also maintains replication fork stability (and hence the S-phase checkpoint) and has been implicated in facilitating homologous recombination repair [10, 12, 13]. As a result of promising early studies, several CHK1 inhibitors have been developed and are currently undergoing clinical evaluation in combination with genotoxic drugs [6, 8, 14-16].

Recent studies have also indicated that CHK1 inhibition alone may have therapeutic activity in certain genetic backgrounds [6, 17, 18]. There is increasing evidence that many tumors harbor substantial amounts of DNA damage as a result of replication stress. This process appears to be intimately associated with tumor development and may arise as a result of oncogene-induced increases in the firing of replication origins. As a consequence, depletion of RPA and dNTPs results in the accumulation of stalled replication forks [19, 20]. This in turn leads to an increased requirement for CHK1 to prevent fork collapse and DNA damage. In support of this hypothesis, single-agent CHK1 inhibitor activity has been demonstrated in several tumor types including MYC-driven tumors such as neuroblastoma and lymphoma as well as acute myeloid leukemia and melanoma, all diseases thought to be associated with high levels of replication stress [17, 18, 21, 22].

We have therefore discovered a novel, potent, orally active CHK1 inhibitor and clinical development candidate: CCT245737. Here we describe the preclinical pharmacology and pharmacodynamics (PD) of this compound together with its therapeutic activity in combination with various genotoxic anticancer drugs in multiple human tumor xenografts. Uniquely, we present clear evidence that the combination of gemcitabine and CCT245737 provides a substantial therapeutic advantage over either agent alone in an antitumor context, thus validating this approach. We describe a novel ELISA for pS296CHK1, which demonstrated target inhibition following CCT245737 treatment at efficacious doses with gemcitabine and carboplatin in a RAS mutant human tumor xenograft model of NSCLC, an area of unmet clinical need in cancer treatment. In addition CCT245737 showed significant antitumor activity as a single-agent in an E*μ-Myc* driven mouse model of B-cell lymphoma. Consequently CCT245737 is in late stage preclinical development for scheduled entry into phase I clinical trials.

## RESULTS

### Structure and kinase selectivity of CCT245737

**Figure 1:**
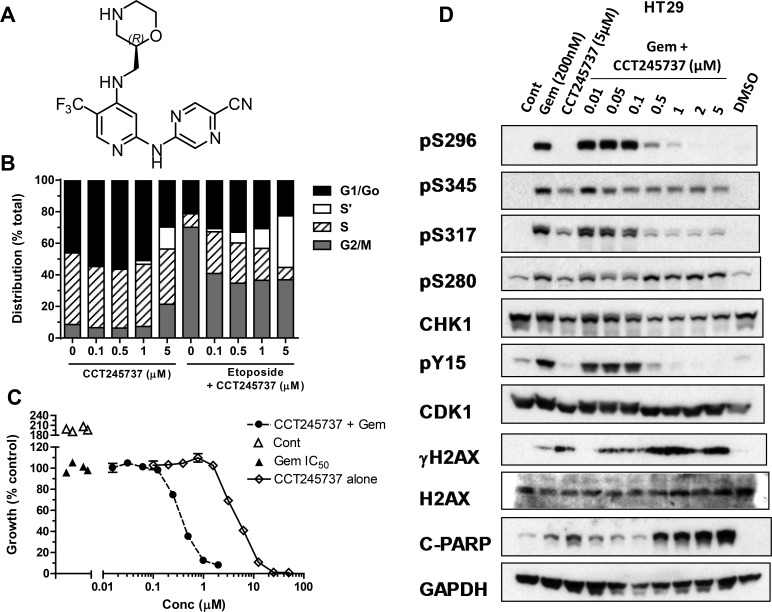
Structure and cellular pharmacology of CCT245737 **A.** Chemical structure of CCT245737 **B.** Effects of 24h exposure to CCT245737 either alone or following acute etoposide exposure (50μM × 1h) on the cell cycle distribution in HT29 colon carcinoma cells. BrdU and PI staining were carried out as described in Materials and Methods. Similar results were obtained in a repeat experiment. **C.** Representative results for a potentiation assay using a fixed concentration of gemcitabine (GI_50_, ▲) and increasing concentrations of CCT245737 either alone ( ◊ ) or in combination with gemcitabine (●) in SW620 colon cancer cells. Vehicle treated cells ( Δ ). Data points are mean±SE for 4 replicates. **D.** Characterization of the effects of CCT245737 on gemcitabine-induced biomarker expression in HT29 colon cancer cells. Protein expression was measured using western blotting as described in Materials and Methods with 50μg sample per lane. GAPDH was used as a loading control. Similar results were obtained in a repeat experiment.

Figure 1A shows the chemical structure of CCT245737 ((*R*)-5-((4-((morpholin-2-ylmethyl)amino)-5-(trifluoromethyl)pyridin-2-yl)amino)pyrazine-2-carbonitrile). A model of CCT245737 bound in the ATP pocket of human CHK1 is shown in Supplementary Figure S1 and suggests that key interactions in the ATP binding site have been retained through hydrogen bonding to Glu85 and Cys87 in the hinge region, the hydrogen bonding of the nitrile to Lys38, while the basic nitrogen on the morpholine forms a salt bridge to Glu91. Similar binding poses have been observed for several other 2-amino-pyrazine-5-carbonitrile CHK1 inhibitors [23].

Initial *in vitro* kinase profiling of CCT245737 (10μM) against 124 kinases showed that only 12 kinases (including CHK1) had > 80% inhibition (Supplementary Table 1). *In vitro* IC_50_ values were determined for these 12 kinases and five others including CDK2/CycA and CDK1/CycB (Supplementary Table 2). CCT245737 was a potent inhibitor of recombinant human CHK1 with IC_50_ of 1.4±0.3nM (mean±SD, *n* = 3, EZ Reader II assay). There was > 1,000-fold selectivity for CHK1 versus the functionally important kinases CDK1 and CHK2 (IC_50_ 1.26-2.44 and 9.03 μM, respectively), and at least a 90-fold selectivity against cross-reacting kinases such as ERK8, PKD1, RSK1 and 2 (see Supplementary Table 2, ^33^P radiometric assay); thus demonstrating that CCT245737 is a potent and selective CHK1 inhibitor.

### Cellular pharmacology of CCT245737

The ability of CCT245737 to abrogate an etoposide-induced G_2_ checkpoint (MIA) in four cell lines is shown in Table 1 and IC_50_ values ranged from 30 to 220nM, confirming potent cellular CHK1 inhibition. There was a greater than 10-fold range in GI_50_ (0.41 to 5.4μM) for single-agent CCT245737 in these cell lines. Cell cycle studies (Figure 1B) showed that CCT245737 had minimal effects in HT29 cells up to 0.5μM for 24h, but at higher concentrations there was an apparent decrease in the G_1_/G_o_ population and a concomitant increase in the S' population, which are unlabelled S-phase cells that may represent a replication crisis [24]. Etoposide alone caused a marked loss of G_1_/G_0_ and a corresponding G_2_/M block. The addition of increasing concentrations of CCT245737 abrogated this G_2_/M block with a corresponding increase in the S'-phase population, confirming that CCT245737 can abrogate an etoposide-induced G_2_/M arrest.

**Table 1:** Summary of *in vitro* MIA, SRB & PI data for CCT245737

Cells	MIA (μM)	GI50 (μM)	Genotoxic Agent	Combination GI50 (μM)	Potentiation Index (PI)
**HT29**	0.030±0.012 (*n* = 6)	0.70±0.29 (*n* = 6)	SN38	0.39±0.061 (*n* = 3)	1.8±0.31* (*n* = 3)
			Gem	0.09±0.023 (*n* = 6)	7.9±2.1*** (*n* = 6)
**SW620**	0.22±0.047 (*n* = 3)	5.4±1.4 (*n* = 5)	SN38	1.8±0.57 (*n* = 4)	3.1±1.5* (*n* = 4)
			Gem	0.33±0.064 (*n* = 7)	17±3.4*** (*n* = 7)
**MiaPaCa-2**	0.063±0.011 (*n* = 3)	1.3±0.40 (*n* = 3)	SN38	0.43±0.062 (*n* = 3)	3.1±0.43* (*n* = 3)
			Gem	0.066±0.025 (*n* = 3)	23±9.6 (*n* = 3)
**Calu6**	0.084±0.0070 (*n* = 3)	0.41±0.059 (*n* = 3)	SN38	0.32±0.021 (*n* = 3)	1.3±0.084* (*n* = 3)
			Gem	0.046±0.0069 (*n* = 3)	9.1±1.5* (*n* = 3)

Mitosis induction assay (MIA IC_50_) results and cytotoxicity (GI_50_) values were determined as described in Material and Methods. Potentiation index (PI) is the ratio of GI_50_ / Combination GI_50_. Potentiation index (PI) values.1 indicate potentiation of the genotoxic drug. Values are mean±SD of n independent determinations.

* Statistical significance: *P* < 0.05

** *P* < 0.01

*** *P* < 0.001.

Previous studies have established that the antimetabolite gemcitabine and topoisomerase 1 inhibitor SN38 are amongst the best candidate drugs for combination with CHK1 inhibitors [24, 25]. Table 1 confirms that in several human tumor cell lines, CCT245737 significantly enhanced the cytotoxicity of these two anticancer drugs with the greatest potentiation occurring with gemcitabine (Figure 1C). Importantly, similar studies using a more conventional approach to determining the potentiation, with minimally toxic concentrations of CCT245737 and a range of genotoxic drug concentrations, showed a similar relationship (Supplementary Figure 2). However, this conventional assay showed less potentiation and was markedly more sensitive to variations in the CHK1 inhibitor concentration employed, which has implications for early drug development as discussed later. There was no statistically significant difference in cytotoxicity, MIA or PI activity of the S-enantiomer CCT252463 compared with CCT245737 (data not shown).

Cellular PD biomarker studies in HT29 colon tumor cells showed that gemcitabine markedly induced autophosphorylation of CHK1 on S296 and phosphorylation of S280, S317 and S345 (Figure 1D). In addition, pY15 CDK1 was increased, consistent with a drug-induced cell cycle arrest. The addition of ≥0.5μM CCT245737 to gemcitabine caused a marked loss of pS296 signal with complete inhibition at ≥2μM CCT245737. Similar effects were seen on pS317 CHK1 but by contrast there was less marked or minimal inhibition of the other CHK1 phosphorylation sites (pS280 and pS345) or alteration in the expression of total CHK1. Loss of pY15 CDK1 corresponded exactly with loss of pS296 CHK1, consistent with abrogation of cell cycle checkpoint and there was a marked co-ordinate increase in pS139 H2AX (γH2AX) a marker of DNA double-strand breaks and cleaved PARP, a marker of apoptosis. Loss of other inhibitory phosphorylations, such as pT14 CDK1 might further enhance the activity of CCT245737. These observations imply that CCT245737 caused CHK1 inhibition, abrogated gemcitabine induced cell cycle arrest causing DNA damage and cell death. Similar results were obtained in the SW620 colon cancer cell line with either gemcitabine or SN38 (Supplementary Figure 3). These data confirm that CCT245737 can inhibit CHK1 activity and enhance the cytotoxicity of gemcitabine and SN38 in several human cancer cells line *in vitro*.

### Pharmacokinetic and pharmacodynamic relationships of CCT245737 in human tumor xenografts in mice

**Figure 2:**
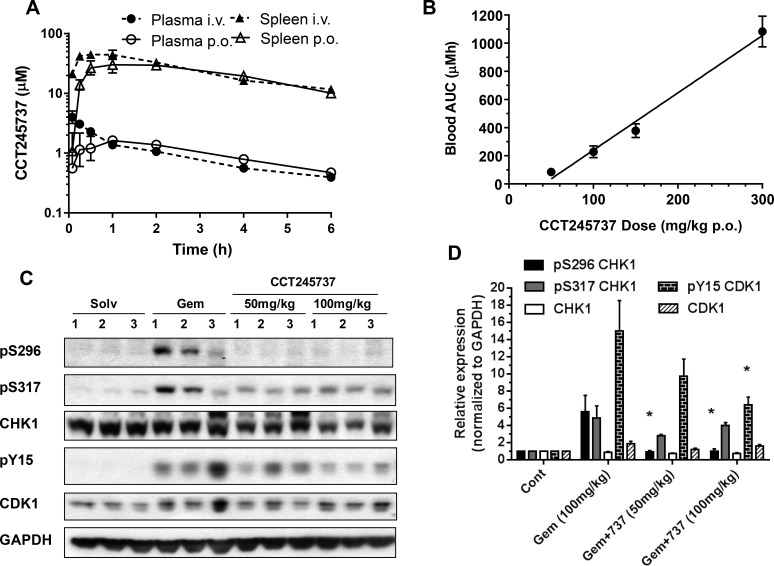
Summary of the pharmacokinetic and pharmacodynamic properties of CCT245737 *in vivo* **A.** Pharmacokinetics of CCT245737 in plasma and spleen following single bolus administration of 10mg/kg either i.v. or p.o. in BALB/c mice. **B.** Relationship between drug exposure (blood AUC) and drug dose for CCT245737 in BALB/c mice. **C.** Pharmacodynamic effects of CCT245737 on gemcitabine-induced CHK1 and cell cycle biomarkers at 24h in human HT29 colon carcinoma xenografts. A single dose of gemcitabine was administered on day 0 (100mg/kg i.v.) and a single dose of CCT245737 administered 24h later (50 or 100mg/kg p.o.) and samples were taken 24h following the last drug dose. Protein expression was measured by western blotting as described in Materials and Methods and GAPDH was used as a loading control. Similar results were obtained at the 6h time point. **D.** Quantification of the pharmacodynamic effects of CCT245737 shown in Figure 2C. Protein expression was quantified using ImageQuant software and normalized to GAPDH expression. Values are mean±SE, *n* = 3 and statistical analysis was by one-way ANOVA with Dunnett's correction; *P < 0.05 indicates a significant difference from gemcitabine treatment alone.

A critical step in successful drug development is characterization of the pharmacokinetics and metabolism of a molecule, to ensure effective tissue drug exposure [26, 27]. Figure 2A and Supplementary Table 3 summarize the pharmacokinetics of CCT245737 in BALB/c mice following either i.v. or p.o. administration. An i.v. dose of 10mg/kg CCT245737 gave a peak plasma concentration of 4μmol/L, with a half-life of 2.86h, an AUC_0-∞_ of 9.96μmol.h/L, a plasma clearance of 2.1L/h/kg and a large volume of distribution (0.19L). The equivalent oral dose gave an almost identical profile with an AUC_0-∞_ of 10.4μmol.h/L showing complete oral bioavailability (F = 105%). Plasma protein binding in nude mice was 75.6% at 10μM CCT245737. In addition, this compound has high cell permeability, as measured by transport across a CaCo2 cell monolayer, with Pe = 25×10^−6^cm/s. Moreover, there was no evidence of asymmetry in the permeability with apical > basal or basal > apical across the monolayer, indicating that the compound is not a substrate for active efflux by PGP. CCT245737 had linear kinetics in whole blood (Figure 2B). Consistent with the large volume of distribution, concentrations of CCT245737 in the spleen were 10 to 25 times greater than in the plasma for both routes of administration and this was reflected in the correspondingly greater AUC_0-∞_ (Supplementary Table 3). Similarly high tumor/plasma ratios were obtained in HT29 xenografts treated with a fixed dose of gemcitabine and different doses of CCT245737 (Supplementary Figure 4). Moreover, 24h following a dose of 12.5mg/kg p.o. CCT245737, mean tumor drug concentrations were ≥ 3μmol/L, a value that would be expected to markedly inhibit cellular CHK1 kinase activity (see MIA IC_50_ Table 1) and gave significant antitumor activity (Supplementary Table 4).

An *in vivo* pharmacokinetic-pharmacodynamic (PK-PD) study in HT29 tumor xenografts taken from mice administered gemcitabine followed by CCT245737 is shown in Figure 2C and 2D. Gemcitabine alone (100mg/kg i.v.) caused marked pS296 CHK1 induction along with pS317 CHK1 and pY15 CDK1. The addition of CCT245737 at 50 or 100mg/kg p.o. significantly reduced the pS296 signal and pY15 CDK1 at the higher dose (*P* < 0.05, Figure 2D) but not pS317, consistent with CHK1 inhibition *in vivo* and the *in vitro* cellular data (see Figure 1D, Supplementary Figure 3). These PK-PD studies show that following oral administration, for a range of drug doses, adequate CCT245737 tumor drug exposure for durable CHK1 inhibition can be achieved.

### Therapeutic activity of CCT245737 combinations in human tumor xenografts

**Figure 3:**
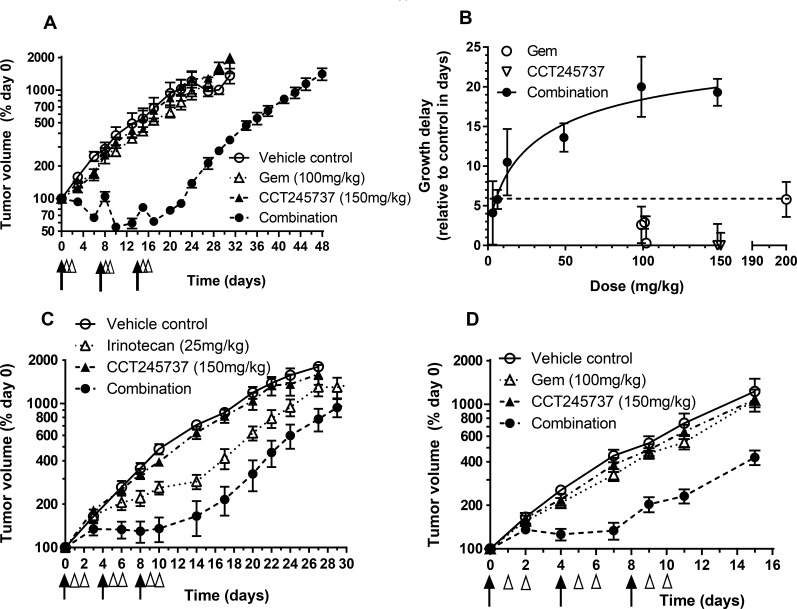
Therapeutic activity of CCT245737 in combination with gemcitabine or irinotecan in human tumor xenografts **A.** CCT245737 enhances the antitumor effects of gemcitabine in HT29 human colon cancer xenografts. Symbols and treatments: o vehicle; Δ gemcitabine alone (100mg/kg i.v. days 0,7 and 14); ▲ CCT245737 alone (150mg/kg p.o. days 1,2; 8,9 and 15,16) and ● combination of gemcitabine + CCT245737 at doses and schedules shown for single agents. Values are mean±SE with 5-6 mice per point. **B.** Summary of the antitumor effects of different oral doses of CCT245737 combined with a fixed dose of gemcitabine (100mg/kg i.v.) in HT29 human colon carcinoma xenografts. The activity of the MTD for single dose gemcitabine (200mg/kg i.v.) and CCT245737 (150mg/kg p.o.) are also shown (dashed line). Growth delays (days) were determined in several independent experiments and are shown as mean±SE for *n* = 6-10 mice per point. **C.** Antitumor efficacy of irinotecan or CCT245737 alone or in combination in HT29 human colon carcinoma xenografts. Symbols and treatments: o vehicle; Δ irinotecan alone (25mg/kg i.p. days 0,4 and 8); ▲ CCT245737 alone (150mg/kg p.o. days 1,2; 5,6; 9 and 10) and ● gemcitabine + CCT245737 combination at times and doses shown for single agents. **D.** Antitumor activity of gemcitabine or CCT245737 alone or combined in SW620 human colon cancer xenografts. Symbols and treatments: o vehicle; Δ gemcitabine alone (100mg/kg i.v. days 0,4 and 8); ▲ CCT245737 alone (150mg/kg p.o. days 1,2; 5,6; 9 and 10) and ● gemcitabine + CCT245737 combination at times and doses shown for single agents. Values are mean±SE, *n* = 5-10 mice per point for **C.** and **D.**. Tumor size and growth delay were determined as described in Materials and Methods. The black arrows in **A.**, **C.** and **D.** indicate when the genotoxic agent was administered and the open arrow heads represent CCT245737 administration with day 0 being the start of therapy of established tumors.

Extensive antitumor studies were carried out using gemcitabine and different doses of CCT245737 in HT29 human colon tumor xenografts (Figure 3A, 3B and Supplementary Table 4). Figure 3A shows that CCT245737 (150mg/kg p.o.) or gemcitabine alone (100mg/kg i.v.) had minimal antitumor activity with growth delays of 0.3±3.4 and −0.6±2.7 days, respectively (mean±SD, Supplementary Table 4). However, the addition of CCT245737 (150mg/kg p.o.) significantly increased the antitumor activity of gemcitabine giving a growth delay of 19.3±1.7 days (mean±SD, *P* < 0.001, Supplementary Table 4). This enhancement was associated with no body weight loss in drug treated animals (Supplementary Figure 5A). Figure 3B and Supplementary Table 4 show the effect of different doses of CCT245737 up to a maximum of 150mg/kg p.o. on the antitumor effects of a fixed dose of gemcitabine (100mg/kg i.v.) in HT29 xenografts. As expected there appears to be a saturable effect of CCT245737 dose on antitumor activity with the greatest effect occurring at around 100mg/kg p.o. At doses up to 50mg/kg p.o. CCT245737 there was a steep increase in the effects of this CHK1 inhibitor on the tumor growth delay response to gemcitabine indicating that low doses of CCT245737 may still have marked therapeutic activity in this combination. These treatments were minimally toxic with a body weight nadir on day 16 and only 2.5% weight loss in the combination arm (Supplementary Figures 5B & 5C). In addition, there was no antitumor effect of CCT245737 alone at the combination maximum tolerated dose (MTD, 150mg/kg p.o.) and only a small increase in antitumor activity of gemcitabine alone at the MTD of 200mg/kg i.v. from 3 to 6 days (Figure 3B and Supplementary Table 4). Importantly, these results clearly show that the combination of CCT245737 with gemcitabine can readily achieve much greater antitumor activity than either agent alone at their respective MTDs. Moreover there was no increase in toxicity thus validating the therapeutic utility of CHK1 inhibitors *in vivo*.

These antitumor studies with CCT245737 were extended into combinations employing other anticancer drugs such as irinotecan in the colon cancer HT29 xenograft model (Figure 3C, Supplementary Table 5). Once again there was no antitumor activity of CCT245737 alone at the combination MTD (1.0±1.0 days growth delay, mean±SD), however, irinotecan had substantial antitumor activity alone at the dose employed with a growth delay of 6.2±4.6 days (mean±SD, *n* = 6). Nevertheless the addition of CCT245737 doubled the growth delay induced by irinotecan to 12.4 days (*P* < 0.05) with a body weight nadir on day 10 of only 2% loss (Supplementary Figure 5D). Studies in SW620 colon cancer xenografts (Figure 3D) showed that CCT245737 and gemcitabine were minimally active as single agents and the combination showed a significantly enhanced antitumor activity compared with gemcitabine alone (7.3±2.1 versus1.5±2.3 days growth delay, *P* < 0.001, Supplementary Table 5) with minimal toxicity (4.3% body weight loss on day 9, Supplementary Figure 5E). These results demonstrate that CCT245737 can significantly enhance the antitumor activity of irinotecan and gemcitabine in a number of different human tumor xenograft models. Furthermore, when tested in HT29 tumors, the activity of gemcitabine combined with CCT245737 was greater than could be achieved at the MTD of either agent alone.

### A novel, quantitative and sensitive biomarker assay for CHK1 activity

**Figure 4:**
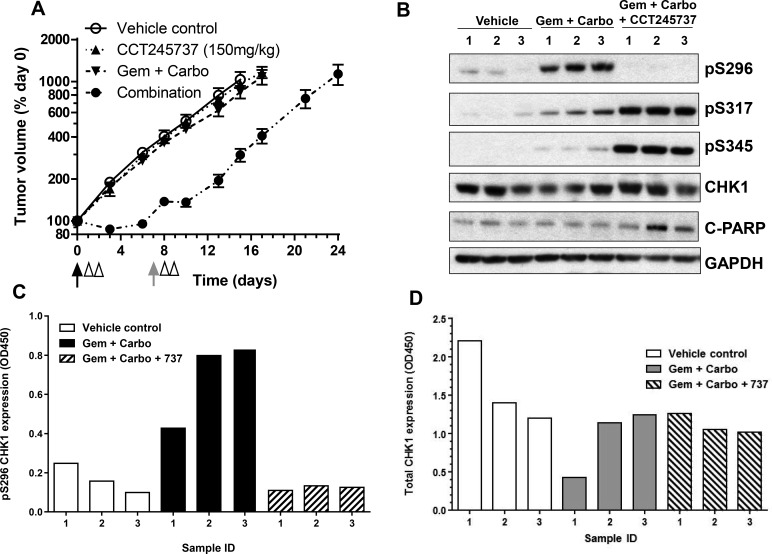
Development and validation of a pharmacodynamic biomarker assay for CCT245737 activity in human lung tumor xenografts **A.** Enhanced activity of gemcitabine and carboplatin combined with CCT245737 compared with either CCT245737 alone (150mg/kg p.o.) or gemcitabine and carboplatin without CCT245737 (100mg/kg i.v. and 5mg/kg i.p., respectively) in Calu6 human lung cancer xenografts. Values are mean±SE, *n* = 6-8 mice per point. The black arrows indicate the time of gemcitabine and carboplatin drug administration, the gray arrow when gemcitabine was administered and the open arrow heads the time of CCT245737 administration. **B.** Western blot of biomarker expression in Calu6 tumor xenografts treated as in **A.**. Samples were taken 6h after the last dose of CCT245737 from individual mice and analysed by western blotting as described in Materials and Methods with GAPDH as a loading control. **C.** Expression of pS296 CHK1 and **D.** total CHK1 expression measured in the same human tumor xenograft samples as shown in **B.** using novel ELISA methods the development of which are described in Methods and Materials and Results.

CCT245737 may be evaluated in several clinical settings, including solid tumors treated with genotoxic drugs, such as lung cancers. This will require a validated assay for a suitable PD biomarker to confirm that CHK1 inhibition has occurred. We therefore developed an ELISA for measuring S296 CHK1 autophosphorylation (pS296) in human tumor material as this is the most sensitive and specific proximal biomarker of CHK1 kinase activity (see Materials and Methods for details). Figure 4A shows that CCT245737 alone or gemcitabine plus carboplatin combined (a standard treatment for lung cancer) had minimal antitumor activity in the Calu6 RAS mutant NSCLC human tumor xenograft model (Supplementary Table 5). The addition of CCT245737 to the genotoxic agents resulted in a statistically significant 9 day increase in tumor growth delay (Supplementary Table 5, *P* < 0.001) with minimal weight loss (nadir on day 3 = 2%, Supplementary Figure 5F). Western blotting for PD biomarker changes in Calu6 xenograft tumors taken from individual mice (Figure 4B) showed that the combination of gemcitabine and carboplatin markedly induced pS296 CHK1 but had minimal effects on pS317 and pS345 CHK1 signals, consistent with our previous studies (Figure 1D [24, 28]). The addition of CCT245737 completely abolished the pS296 signal but actually enhanced both pS317 and pS345 CHK1 levels - confirming once again that pS296CHK1 is a sensitive, robust and reproducible biomarker of CHK1 inhibition. There were minimal changes in total CHK1 expression. Using the same Calu6 tumor lysates from these studies we show that the ELISA assay for pS296 CHK1 accurately reproduced this signal as detected by western blotting (Figure 4C). In addition the ELISA for total CHK1 showed minimal changes consistent with the immunoblotting results (Figure 4D). Both assays were capable of detecting recombinant pS296 and total CHK1 down to a concentration of 0.4nM (assuming a signal:noise ratio of 2:1). These results show that we have developed an accurate and sensitive ELISA for pS296 CHK1, a biomarker of CHK1 activity, which can be quantified in human tumor tissue.

### Single-agent antitumor activity of CCT245737 in an E*μ-Myc* driven mouse model of B-cell lymphoma

**Figure 5:**
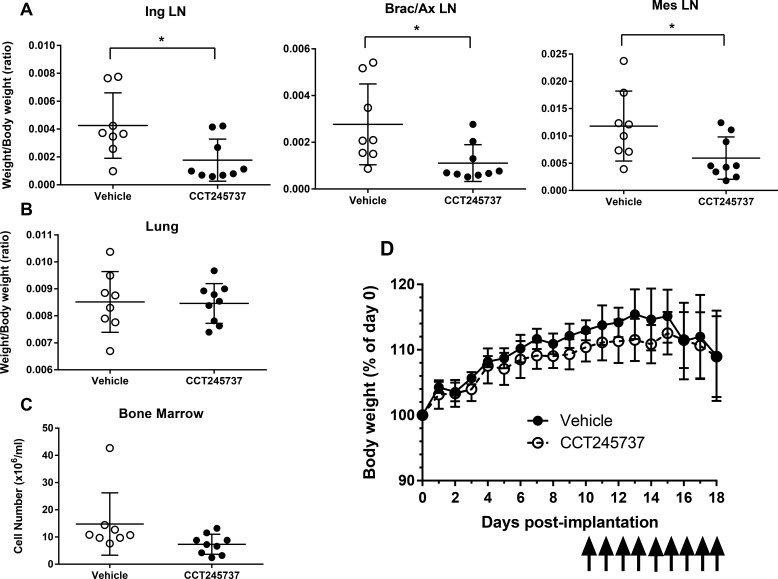
Antitumor activity of single-agent CCT245737 in an E*μ-Myc* mouse model of human B-cell lymphocytic leukemia **A.** Effect of CCT245737 treatment on: inguinal (Ing), brachial/axillary (Brac/Ax) and mesenteric (Mes) lymph node (LN) mass compared with controls in E*μ-Myc* tumor transplanted mice. These sites are commonly associated with infiltrating B-cell lymphoma in this model system. **B.** Effect of CCT245737 treatment on normal lung tissue mass and **C.** bone marrow cell number in E*μ-Myc* tumor transplanted mice. **D.** Effect of CCT245737 treatment on mouse body weight. Animals were administered CCT245737 (150mg/kg p.o.) or vehicle, once daily for 9 days (as indicated by arrows) starting 10 days post-implantation. Mice were culled 24h after the last treatment. Values are mean±SD, *n* = 7-9 and statistical analysis was by Students t-test: *P < 0.05 indicates a significant difference from vehicle control treated animals.

The deregulation of certain oncogenes such as MYCN in neuroblastoma and C-MYC in lymphoma appears to be associated with an increase in single-agent CHK1 sensitivity, possibly through enhanced replication stress. We therefore evaluated the antitumor activity of CCT245737 in an E*μ-Myc* driven transgenic mouse transplant model of B-cell lymphoma that infiltrates the lymph glands. Figure 5A clearly shows that 9 days oral administration of 150mg/kg CCT245737 significantly reduced the weight of the inguinal, brachial/axillary and mesenteric lymph nodes compared with vehicle treated control animals (*P* < 0.05). Moreover there were minimal effects on normal tissues such as the lung (Figure 5B, *p* = 0.904) and bone marrow (Figure 5C, *p* = 0.0838) or kidney (*p* = 0.959, not shown). There was a significant decrease in thymus weight following CCT245737 treatment (*p* < 0.01) suggesting some tumor involvement and spleen weight was increased significantly (*p* < 0.01) suggesting some splenomegaly (not shown). The treatment was well tolerated as evidenced by stable body weights (Figure 5D) and negligible effects on other parameters such as water consumption and body temperature (data not shown). Consequently these results show that CCT245737 has significant antitumor activity as an oral single-agent in a *Myc*-driven mouse model of B-cell lymphoma.

## DISCUSSION

CCT245737 is a novel, potent, selective and well-tolerated orally active CHK1 inhibitor, and our clinical development candidate. Enzyme kinetic and modeling data clearly show that CCT245737 is a potent, ATP-competitive inhibitor of human CHK1 kinase with an IC_50_ of 1.4nM. Initial screening against other kinases demonstrated an appropriate selectivity profile with subsequent studies showing ≥ 500-fold selectivity for all but five kinases. Importantly there was minimal inhibition of CHK2 which has little therapeutic utility in genotoxic combinations [29, 30]. There was also no cross-reactivity with CDK1 and 2, inhibition of which might antagonize the cytotoxic effects of CHK1 inhibition, as CDK1 activation has been implicated in CHK1 inhibitor-based cell killing [30]. This selectivity profile compares favorably with other CHK1 inhibitors, such as AZD7762 [31], GDC-0575 [32], PF00477736 [33] and LY2603618 [34]. There was evidence of potent intracellular CHK1 inhibition in several human tumor cell lines following genotoxic stress (Table 1) and this was associated with abrogation of a late S, G_2_/M checkpoint (Figure 1C) consistent with CHK1 inhibition and similar to other CHK1 inhibitors [28, 33]. Several previous studies have established that certain classes of genotoxic anticancer drugs are potentiated more effectively than others by CHK1 inhibitors e.g. antimetabolites and topoisomerase 1 inhibitors [25, 35].

Using a rapid and sensitive assay for determining the combination GI_50_ (where a fixed concentration of the genotoxic agent is combined with a range of CCT245737 concentrations) we derived a potentiation index (ratio of IC_50_ for CCT245737 alone: combination GI_50_) and showed that the cytotoxicity of both gemcitabine and SN38 were significantly enhanced (Table 1). This technique was validated by comparison with the more conventional approach where a non-toxic concentration of a CHK1 inhibitor is combined with increasing concentrations of the genotoxic drug (Supplementary Figure 3) with qualitatively similar results. The rapid technique presented here has the advantage of quickly establishing a genotoxic GI_50_ in contrast to the most active non-toxic CHK1 inhibitor concentration. Moreover, this rapid technique was more sensitive than the conventional approach with 2 to 3-fold greater PI values, allowing evaluation and ranking of relatively non-potent and selective compounds in the early stages of the CHK1 inhibitor drug discovery project.

It is clear from these and other available data that antimetabolites such as gemcitabine are potentiated by several CHK1 inhibitors to a greater extent than topoisomerase 1 inhibitors [24, 25, 35]. This may reflect a greater ability of the former to deplete nucleotide pools and enhance replication stress with an increased reliance on CHK1 to avoid replication fork collapse [36, 37]. Our cellular PD biomarker studies reproducibly showed that there was a concentration-dependent inhibition of genotoxic induced pS296 CHK1 autophosphorylation by CCT245737. Moreover this pS296 CHK1 signal was a more sensitive and robust readout of CHK1 inhibition in our systems than other phospho-CHK1 sites. This is in contrast to other reports [38], possibly due to the fact that pS296 is a specific and direct readout of CHK1 activity [39], an essential requirement for PD biomarkers of kinase inhibitors. This CCT245737 concentration-dependent loss of pS296 CHK1 signal was coincident with loss of cell cycle arrest, increased DNA damage and apoptosis implying that CHK1 inhibition induced cell death [3, 40].

Pharmacokinetic studies with CCT245737 showed complete oral bioavailability with linear pharmacokinetics and high tumor/plasma ratios consistent with extensive tumor exposure. Pharmacodynamic studies showed that CCT245737 concentrations required for inhibition of gemcitabine-induced pS296 CHK1 and pY15 CDK1 were readily achieved in tumors at 24h following a single oral dose of 50mg/kg (Figure 2C). We and others have shown that an effective antitumor combination strategy involves delaying CHK1 inhibitor administration by 24h following a genotoxic drug dose and then extending effective CHK1 exposure for a further 48h [7, 24, 35]. Moreover there were no detectable pharmacokinetic interactions with this CHK1 inhibitor chemical class and the genotoxic agents studied. The PK-PD relationship described here for CCT245737 and its high oral bioavailability, make this an ideal compound for clinical evaluation. `

Efficacy studies in a number of different xenograft tumor models showed that CCT245737 enhanced the activity of both gemcitabine and irinotecan. Importantly and uniquely, we show for the first time that a CHK1 inhibitor can markedly enhance the antitumor activity of gemcitabine to a greater extent than can be achieved by the MTD of either agent alone. Moreover the dose-response curve had a relatively steep initial phase, suggesting that a therapeutically useful increase in gemcitabine activity is feasible at modest doses of CCT245737, as might be achieved in early stage clinical trials. This response appeared to plateau at doses above 50mg/kg, implying that very high concentrations of CCT245737 may not be required for optimal therapeutic activity and consistent with the idea that CHK1 inhibition in tumors may enhance DNA damage or stalled replication fork collapse [8, 13]. These observations further support the clinical development and evaluation of CCT245737 in combination with genotoxic agents such as gemcitabine.

To this end we have shown that CCT245737 can significantly enhance the antitumor activity of both gemcitabine and carboplatin in a *RAS* mutant NSCLC human tumor xenograft model. This is an area of unmet clinical need and a disease setting in which clinical testing of CCT245737 is proposed. In order to ensure that these antitumor effects are a result of CHK1 inhibition we also carried out PD studies and showed once again that pS296 CHK1 inhibition is a more sensitive, robust and reproducible marker of CHK1 inhibition than either pS317 or pS345 CHK1. Indeed, induction/inhibition of pS317 and pS345 CHK1 by CCT245737 appeared to be either concentration and/or time dependent (Figure 1D and Supplementary Figure 3) as well as context dependent (Figure 2C and 4B) possibly through differences in feedback repression of ATR/ATM, DNA damage and repair capacity or PP2A and WIP1 phosphatase activity. Consequently we developed a novel, sensitive and quantitative ELISA for pS296 CHK1 which will facilitate the clinical evaluation of this combination and allow target inhibition monitoring in the patient. Nevertheless other indirect readouts such as CDC25A or pY15 CDK1 loss, apoptosis markers and γH2AX and RAD51 foci formation may prove useful in confirming that functionally significant CHK1 inhibition has occurred [8, 24, 41, 42].

An intriguing aspect of CHK1 inhibitor development is the realization that these compounds may exhibit single-agent activity in particular malignancies. It appears that tumors with deregulated MYC expression or high levels of replication stress are very sensitive to single-agent CHK1 inhibition [17, 18, 21, 22]. To expand the potential clinical utility of CCT245737 monotherapy we have shown that it has significant antitumor activity in an E*μ-Myc* driven transgenic murine model of infiltrating B-cell lymphoma (Burkitt's-type lymphoma) [43]. MYC gene alterations are common in other B-cell neoplasms and are often associated with poor outcomes [44]. CCT245737 as a single-agent was well tolerated in the E*μ-Myc* transgenic model and had minimal effects on normal tissues such as lung and bone marrow and kidney, although there was some evidence of splenomegaly. However the use of an isogenic MYC inducible model would be even more definitive. Nevertheless, these results are consistent with studies using the CHK1 inhibitor PF-0477736 in a variety of E*μ-Myc* driven transgenic murine lymphoma cell lines [18] and are directly comparable with antitumor studies using UCN01 (5mg/kg × 9 days), a non-selective CHK1 inhibitor in the same model [45]. In the latter case clear evidence of tumor growth inhibition was associated with a decreased blood cell count, possibly due to off-target effects of UCN01 and in marked contrast to this study. Should CHK1 inhibitors prove successful in the clinic it will be important to identify biomarkers of sensitivity to facilitate patient selection and to establish other molecular targets, which upon inhibition give a synthetically lethal phenotype [46].

In conclusion, we have investigated the preclinical pharmacology, PK-PD relationships and antitumor activity of the novel, potent, selective and orally active CHK1 clinical development candidate CCT245737. We clearly show that CCT245737 enhanced the antitumor activity of several anticancer agents *in vivo* and uniquely demonstrate that for gemcitabine this was greater than the activity achievable by dose escalation of either agent alone. The lack of additional toxicity in the combinations thus provides a valuable therapeutic gain. We also show that pS296 CHK1 is a robust PD biomarker of CHK1 activity and provide a novel, quantitative ELISA-based assay suitable for the measurement of this biomarker in clinical material. We demonstrate that CCT245737 has single-agent antitumor activity in a *Myc* driven model of B-cell lymphoma, providing preclinical evidence of a potential adult clinical monotherapy setting. Consequently CCT245737 is in late stage preclinical development in preparation for a first-in-man phase I clinical trial.

## MATERIALS AND METHODS

### Drugs and compounds

CCT245737 (Figure 1A) and its S-enantiomer CCT252463 were synthesized as described [47]. Irinotecan was purchased from Pfizer and its active metabolite (SN38) from LKT laboratories. Gemcitabine was obtained from Eli Lilly. All other compounds were purchased from Sigma.

### Human tumor cell lines

The colon tumor cell lines HT29 (p53^−/−^, APC^+/−^, PIK3CA^+/−^, BRAF^+/−^ and SMAD4^−/−^) and SW620 (p53^−/−^, KRAS^−/−^, APC^−/−^, SMAD4^−/−^ and MAP2K4^−/−^), the pancreatic cancer cell line MiaPaCa-2 (p53^−/−^, KRAS^+/−^, KDM6A^−/−^, CDKN2a; p14^−/−^ and CDKN2A) and the non-small cell lung cancer Calu6 cell line (p53^−/−^ and KRAS^+/−^) were purchased from ATCC (Lot numbers 4487729, 3924081, 57866607 and 58683029, respectively and Sanger Centre data http://www.sanger.ac.uk/genetics/CGP/cosmic). Cells were grown in Dulbecco's modified Eagles medium containing 10% fetal calf serum and 2mM glutamine and were mycoplasma free [24].

### *In vitro* kinase assays

Commercial *in vitro* ^33^P radiometric kinase assays were carried out against 124 human kinases using 10μM CCT245737 at ATP concentrations corresponding to the kinase K_m,ATP_ (MRC Phosphorylation Unit, Dundee). Other kinase IC_50_ determinations for CHK2 and FLT3 were performed using a commercial assay (Z'-Lyte, Invitrogen) or in-house with recombinant human CHK1 on a LabChip^®^ EZ Reader II (PerkinElmer) or CDK1 in a DELFIA assay (PerkinElmer).

### Cellular cytotoxicity, G_2_ checkpoint abrogation and potentiation assays

Assays were performed as described [24]. Cytotoxicity was determined as the drug concentration that gave 50% inhibition of tumor cell proliferation (GI_50_) using a 96h (i.e. 4-doublings) Sulforhodamine B (SRB) assay. Inhibition of intracellular CHK1 activity was measured using a cell based ELISA for the abrogation of an etoposide induced G_2_ checkpoint (mitosis induction assay, MIA [24]). The IC_50_ for G_2_ checkpoint abrogation (MIA) was determined in the presence of nocodazole using UCN01 as a positive control. The activity index (AI) was used as a measure of the compounds ability to induce mitosis relative to its toxicity (i.e., ratio of MIA IC_50_: 96h SRB GI_50_). Routine potentiation studies were carried out using a fixed concentration (GI_50_) of either gemcitabine or SN38 in combination with a range of CCT245737 concentrations to determine the combination GI_50_ of CCT245737. The ability of CCT245737 to enhance gemcitabine or SN38 cell killing was expressed as a potentiation index (PI) equal to the ratio of the GI_50_ for CCT245737 alone versus the combination GI_50_ for CCT245737 (see above). PI values > 1 indicate potentiation of the genotoxic activity. In addition, a series of experiments was carried out using fixed, non- or minimally toxic concentrations of CCT245737 (≤GI_20_) with a range of different concentrations of gemcitabine or SN38 to determine the extent to which CCT245737 enhanced drug cytotoxicity compared with the genotoxic agent alone, i.e. conventional PI (ratio GI_50_ genotoxic alone: GI_50_ genotoxic combined with non-toxic CCT245737 concentration, Con PI).

### Immunoassays

Western blotting was carried out as described [24]. ELISA assays for pS296 CHK1 and total CHK1 were developed and validated for use *in vitro* and in tumor homogenates. Tumor samples for ELISA and western blotting were lysed in CHAPS buffer (1% CHAPS in 150mM NaCl, 50mM Tris-HCl pH 8.0, 1mM EDTA, 0.2mM PMSF and 1:50 Sigma Phosphatase inhibitors 2 and 3 and 1:100 Sigma protease cocktail) and immunoblotted. ELISA assays were based on commercially available kits with modifications (Cell Signalling Technologies). For pS296 CHK1 detection the commercial pS317 CHK1 antibody was replaced with a pS296 CHK1 antibody (Cell Signalling Technology). Xenograft tumor homogenate was added in duplicate to 96 well plates (100μl containing 40ug protein) and quantification of the colorimetric readout was determined at 450nm using a PerkinElmer 2103 EnVision Multilabel plate reader.

### Cell cycle analysis

Cell cycle analysis using propidium iodide and bromodeoxyuridine staining was as described [28].

### Animal studies

Compound tolerability and pharmacokinetic investigations were carried out in female BALB/c mice (Charles River). Human tumor xenografts were established s.c. in female CRTac:Ncr-*Fox1(nu)* athymic mice and treated as previously described [24, 28]. The vehicle for oral administration of CCT245737 was DPTW (10% DMSO, 20% PEG400, 5% Tween 80 and 65% water) and gemcitabine and irinotecan were administered in their respective clinical vehicles. Treatments were generally initiated when tumors reached a mean diameter of 5-6mm (day 0). For combination studies, CCT245737 was given orally 24 and 48h after genotoxic drug administration, previously determined as an optimal schedule for CHK1 inhibitor and genotoxic drug combinations [24]. In HT29 xenograft studies, gemcitabine was administered at 100mg/kg i.v. on days 0,7 and 14 and CCT245737 at the indicated doses on days 1,2,8,9,15 and16. Irinotecan was administered at 25mg/kg i.p. on days 0,4 and 8 with CCT245737 administered at 150mg/kg p.o. on days 1,2,5,6,9 and 10. In SW620 and Calu6 xenograft studies, gemcitabine was administered at 100mg/kg i.v. on days 0,4 and 8 and CCT245737 subsequently at 150mg/kg on days 1,2,5,6,9 and 10. For the Calu6 xenograft studies involving gemcitabine and carboplatin, drugs were administered at 100mg/kg i.v. and 5mg/kg i.p., respectively on day 0 with gemcitabine alone at 100mg/kg i.v. on day 7 with CCT245737 at 150mg/kg p.o. on days 1,2,8 and 9. The genotoxic drug doses employed were sub-maximally active to facilitate detection of subsequent potentiation. Initial treatment groups contained from 6 to 10 mice and animals were inspected daily and tumor size and volume measured every 2 or 3 days. Tumor volume and growth delay were determined as previously described [28].

Transgenic E*μ-Myc* mice which develop aggressive infiltrating lymphoma were established and monitored as previously described [48, 49]. To generate transgenic E*μ-Myc* driven lymphoma allografts, tumor cells from 3 separate tumors were harvested, cells counted and injected via a tail vein. Six mice were set up per tumor to provide 3 control and 3 treated animals, giving a maximum of 9 mice per treatment group. Animals were monitored daily and continuously using RFID transponders to measure temperature, activity and water consumption as previously described [49]. For studies of single-agent CCT245737 activity in mice injected with transgenic E*μ-Myc* lymphoid tumor cells, CCT245737 was administered at 150mg/kg p.o. for 9 successive days with culling 24h after the last dose. Lymph nodes and other tissues were removed from vehicle and CCT245737 treated mice and their weights and tissue/body weight ratios compared to assess antitumor activity. Bone marrow cellularity was also determined to check for tumor cell involvement. All mice were handled in compliance with local and national animal welfare guidelines [50].

### Pharmacokinetics

Compounds were extracted from whole blood, plasma and tissue homogenates with methanol-containing internal standards using established protocols. Drug concentrations were determined using liquid chromatography/tandem mass spectrometry (LC/MS-MS) and pharmacokinetics were calculated using Pharsight WinNonLin software (version 5.2.1) as previously described [28].

### Statistics

Statistical significance (*, *P* < 0.05; **, *P* < 0.01; ***, *P* < 0.001) was determined using an unpaired, one-tailed or two-tailed *t*-test or one-way ANOVA with either Tukey or Dunnett test, as appropriate using GraphPad Prism 5 software.

## SUPPLEMENTARY MATERIAL FIGURES AND TABLES


