# Supplementary material for: The clinical development candidate CCT245737 is an orally active CHK1 inhibitor with preclinical activity in RAS mutant NSCLC and Eμ-MYC driven B-cell lymphoma
Source: Oncotarget. 2015 Jul 22;7(3):2329–42. doi: 10.18632/oncotarget.4919 (PMC4823038; doi:10.18632/oncotarget.4919)
Supplement: Supplementary file 1 [file oncotarget-07-2329-s001.pdf]

The clinical development candidate CCT245737 is an orally active CHK1 inhibitor with preclinical activity in RAS mutant NSCLC and Eμ-MYC driven B-cell lymphoma

Supplementary Material

Supplementary Material

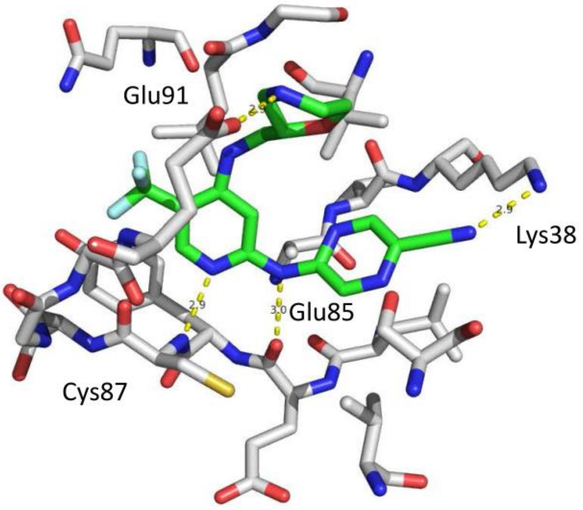

S Fig 1. A constrained scaffold docking model of CCT245737 binding in the ATP pocket of human CHK1. This model was based on the crystal structure of SAR-020106 binding in the CHK1 ATP pocket (PDB 2ym8) [25].

Supplementary Table 1: Initial *in vitro* kinase screen for CCT245737 (MRC Dundee)

MKK1	68	MNK2	75	IKKb	91	CSK	82
MKK2	61	MAPKAP-K2	94	IKKe	102	YES1	65
MKK6	104	MAPKAP-K3	114	TBK1	71	ABL	93
ERK1	75	PRAK	71	PIM1	56	BTK	72
ERK2	98	CAMKKb	73	PIM2	88	JAK2	37
JNK1	104	CAMK1	69	PIM3	36	SYK	84
JNK2	107	SmMLCK	84	SRPK1	82	ZAP70	93
JNK3	106	PHK	15	EF2K	98	TIE2	104
p38a MAPK	120	DAPK1	48	EIF2AK3	105	BRK	108
p38b MAPK	90	CHK1	4	HIPK1	77	EPH-A2	76
p38g MAPK	95	CHK2	15	HIPK2	92	EPH-A4	71
p38d MAPK	54	GSK3b	47	HIPK3	124	EPH-B1	111
ERK8	4	CDK2-Cyclin A	23	CLK2	6	EPH-B2	102
RSK1	7	PLK1	103	PAK2	96	EPH-B3	93
RSK2	19	Aurora A	75	PAK4	49	EPH-B4	81
PDK1	76	Aurora B	26	PAK5	70	FGF-R1	33
PKBa	93	TLK1	59	PAK6	77	HER4	110
PKBb	100	LKB1	44	MST2	48	IGF-1R	104
SGK1	87	AMPK	32	MST4	44	IR	102
S6K1	52	MARK1	43	GCK	64	IRR	109
PKA	93	MARK2	21	MINK1	44	TrkA	31
ROCK 2	95	MARK3	13	MEKK1	77	VEG-FR	13
PRK2	67	MARK4	39	MLK1	75	TAK1	48
PKCa	82	BRSK1	12	MLK3	23	IRAK1	38
PKCγ	87	BRSK2	35	TESK1	101	IRAK4	73
PKCz	92	MELK	37	TAO1	54	RIPK2	101
PKD1	17	DYRK1A	41	ASK1	80	OSR1	109
STK33	39	DYRK2	61	TSSK1	53	TTK	36
MSK1	30	DYRK3	24	CK1	85	MPSK1	122
MNK1	62	NEK2a	96	CK2	81	Src	93
NUAK1	6	NEK6	109	TTBK1	82	Lck	78

Screen was carried out against 124 kinases at 10μM CCT245737 as described in Materials and Methods . Values are % remaining activity

Supplementary Table 2: *In vitro* selectivity of CCT245737 against selected kinases

**Kinase**	**% inh @ 10 μM Dundee**	**IC_{50} (nM) Invitrogen unless stated**	**Approx Fold selectivity**
CHK1	96%	1.4±0.3 (Mean±SD, n=3, in house, Caliper)	-
ERK8	96%	130 (Dundee)	93x
PKD1	83%	298 (PRKD1/PKC-μ)	213x
RSK1	93%	362	258x
RSK2	81%	361	258x
VEG-FR	87%	15800 (VEGFR1); 2110 (VEGFR2)	1500-11300x
NUAK1	94%	711	507x
MARK3	87%	698	499x
CLK2	94%	1370	978x
BRSK1	88%	1660	1190x
CHK2	85%	2440; 1260 (in house)	1320x
AMPK	68%	2970 (AMPK A1/B1/G1)	2120x
PHK	85%	3470 (PHKG1)	2480x
CDK2/CyclA	77%	3850	2750x
FLT3	-	582	416x
CDK1/CyclB	-	9030	6450x

Inhibitory activity of CCT245737 (IC_{50}) was determined using the assays as shown and described in Materials and Methods

Supplementary Figure 2: Determination of conventional potentiation index for CCT245737 *in vitro*

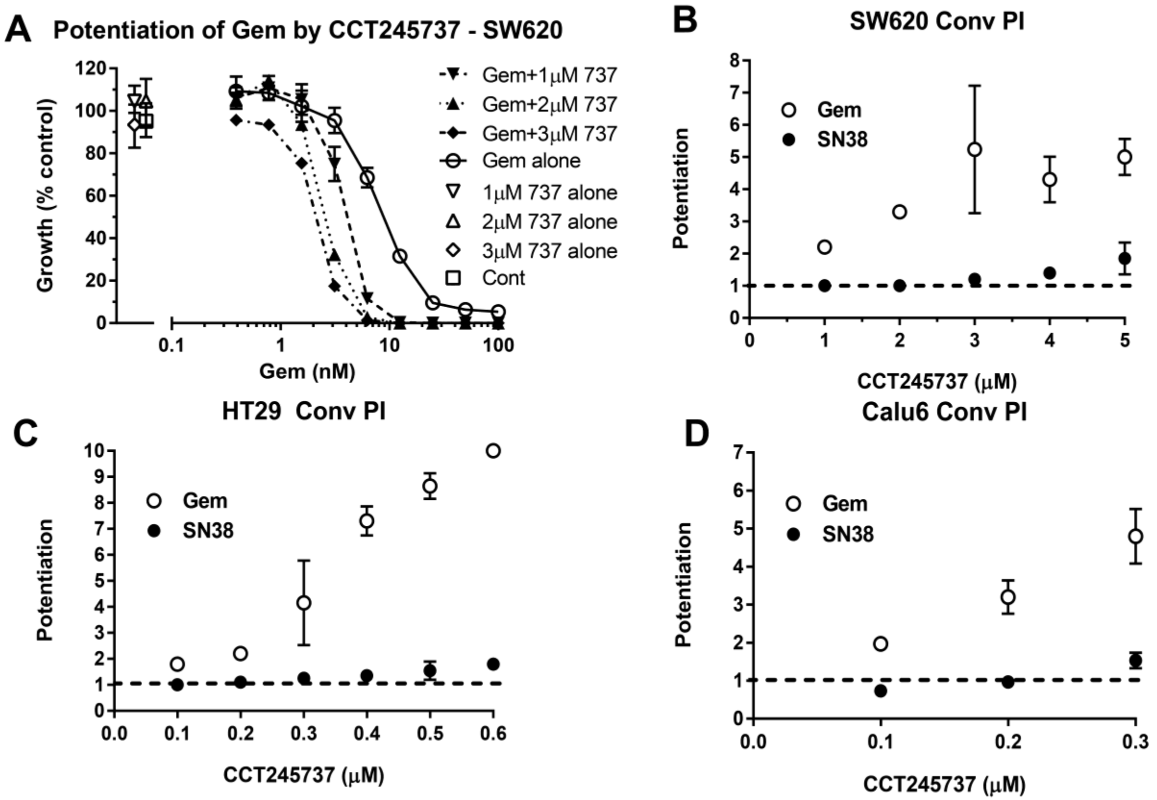

S Fig 2. Determination of conventional potentiation index for gemcitabine and SN38 combined with CCT245737 *in vitro*. A) Effect of a range of minimally cytotoxic concentrations of CCT245737 (1, 2 or 3μM) on gemcitabine cytotoxicity in SW620 human colon cancer cells. Minimal cytotoxicity was defined as ≤ 20% cell kill compared with untreated controls. B) Effect of a range of CCT245737 concentrations on the ability to potentiate gemcitabine and SN38 cytotoxicity in SW620 human colon cancer cells. C) Similar data to B) but showing the ability of CCT245737 to potentiate gemcitabine and SN38 activity in HT29 colon cancer cells. D) Effect of minimally toxic concentrations of CCT245737 on the ability to potentiate gemcitabine and SN38 cytotoxicity in Calu6 human lung cancer cells. Potentiation was determined as the ratio of gemcitabine GI_{50}: gemcitabine + CCT245737 GI_{50}. Minimally cytotoxic concentrations of CCT245737 were defined as ≤20% cell kill compared with untreated controls. Values shown are mean and range for n=2 or SD for n>2.

Supplementary Figure 3: Effect of CCT245737 on genotoxic drug induced cell cycle biomarkers

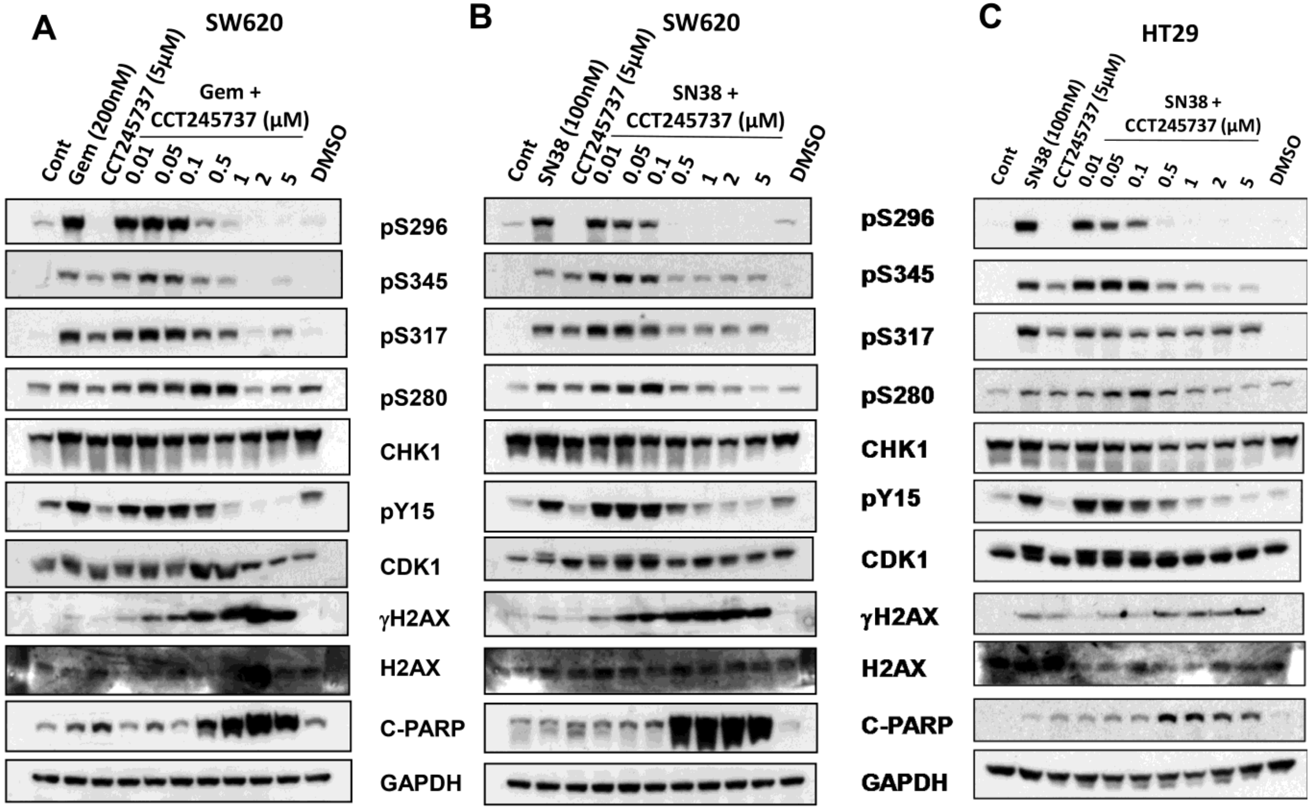

S Fig 3. Effects of CCT245737 exposure for 24h on genotoxic drug-induced CHK1 and cell cycle PD biomarkers in human colon cancer cell lines. A) SW620 cells treated with gemcitabine (200nM) and a range of CCT245737 concentrations. B) SW620 cells treated with SN38 (100nM) and increasing concentrations of CCT245737. C) HT29 cells treated as in B. Cells were pre-treated with CCT245737 for 1h prior to cytotoxic drug exposure. Protein expression was determined by western blotting (50µg lysate per lane) as described in Materials and Methods. CHK1 pS296 autophosphorylation was used as a read-out of CHK1 activity, pY15 CDK1 as a read out of cell cycle inhibition and pS139 (γ) H2AX and cleaved PARP as a biomarkers of DNA damage and apoptosis, respectively. GAPDH was used as a loading control.

Supplementary Table 3

The pharmacokinetic parameters of CCT245737 in BALB/C mice following 10mg/kg i.v. or p.o.

Tissue	Route	Tmax (h)	Cmax (µmol/L)	AUC_{0-t} (µmol.h/L)	$AUC_{0-\infty}$ (µmol.h/L)	T1/2 (h)	Cl (L/h/kg)	V_{ss} (L)	F
Plasma	IV	0.083	4.00	9.94	9.96	2.86	2.12	0.19	
Plasma	PO	1	1.62	10.4	10.4	2.85	2.16		1.05
Spleen	IV	0.5	44.7	262	262	2.76			
Spleen	PO	1	30.1	222	223	3.11			

Non-compartmental analysis determined with WinNonlin version 3.2.1. Values are composite data obtained from 7-8 time points with 2-3 mice per point.

Supplementary Figure 4: Plasma and HT29 tumor CCT245737 concentrations at 24h

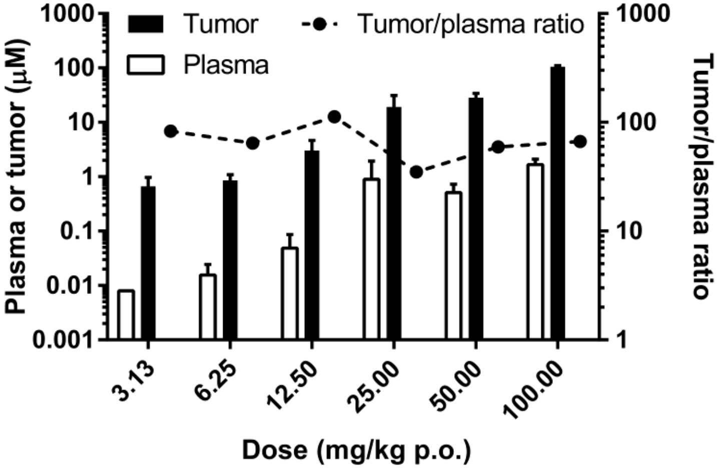

S Fig 4. Concentrations of CCT245737 in plasma and HT29 tumor xenografts 24h following treatment with gemcitabine (100mg/kg i.v.) and different oral doses of CCT245737 in mice. Tumor/plasma ratios are also shown. Values are mean±SD for 3 to 5 animals. Drug concentrations were determined by LC-MS-MS as described in Materials and Methods.

Supplementary Table 4:

Summary of gemcitabine and CCT245737 activity in HT29 human tumor xenografts

Expt	Tumour	Growth		Growth delay (days)		
	(ANOVA)	Cont (days)	Gem	CCT245737	Dose (mg/kg)	Combination
62	HT29 (***)	10.5±5.2	0.3±3.4	-0.6±2.7	150	19.3±1.7***
95	HT29 (***)	8.4±1.6	2.6±2.3	-0.7±1.6	100	20.0±3.8***
					50	13.6±1.8***
11	HT29 (***)	7.2±1.8	2.9±0.8	-1.3±1.2	12.5	10.5±4.2***
					6.3	5.8±1.2
					3.1	4.1±4.0

Statistical significance was determined using a one-way ANOVA with Dunnett's multiple comparison test. Statistics: *. P<0.05; **, P<0.01; ***P<0.001, significantly different from gemcitabine treatment alone. Gem dose was 100mg/kg i.v. Growth was the time taken for controls to reach 300% initial tumor volume. Growth delay values are mean±SD, n = 6-8 animals per experiment and were determined as described [25].

Supplementary Table 5:

Summary of CCT245737 activity in human tumor xenografts

Expt	Tumour	Growth (days)		Growth Delay (days)			
	(ANOVA)	Cont	Irin	Gem	Gem+ Carbo	CCT245737	Combination
96	HT29 (**)	6.8±1.5	6.2±4.6			0.95±1.0	12.4±4.7*
62	HT29 (***)	10.5±5.2		0.3±3.4		-0.6±2.7	19.3±1.7***
94	SW620 (***)	5.0±1.0		1.5±2.3		0.6±1.8	7.3±2.1***
37	Calu6 (***)	6.4±2.5			0.7±2.7	-0.3±1.2	9.7±3.0***

Doses: Irinotecan 25mg/kg i.p. (Irin); Gemcitabine 100mg/kg i.v. (Gem); Carboplatin 5mg/kg i.p. (Carbo) and CCT245737 150mg/kg p.o. (737). Statistical significance was determined using a one-way ANOVA with Dunnett's multiple comparison test. Statistics: *, P,0.05; **, P<0.01; *** P<0.001, significantly different from the cytotoxic treatment alone. Growth was the time taken for controls to reach 300% initial tumor volume. Growth delay values are mean±SD, n = 6-8 animals per experiment and was determined as described [25].

Supplementary Figure 5: Effect of drug treatments on animal body weights

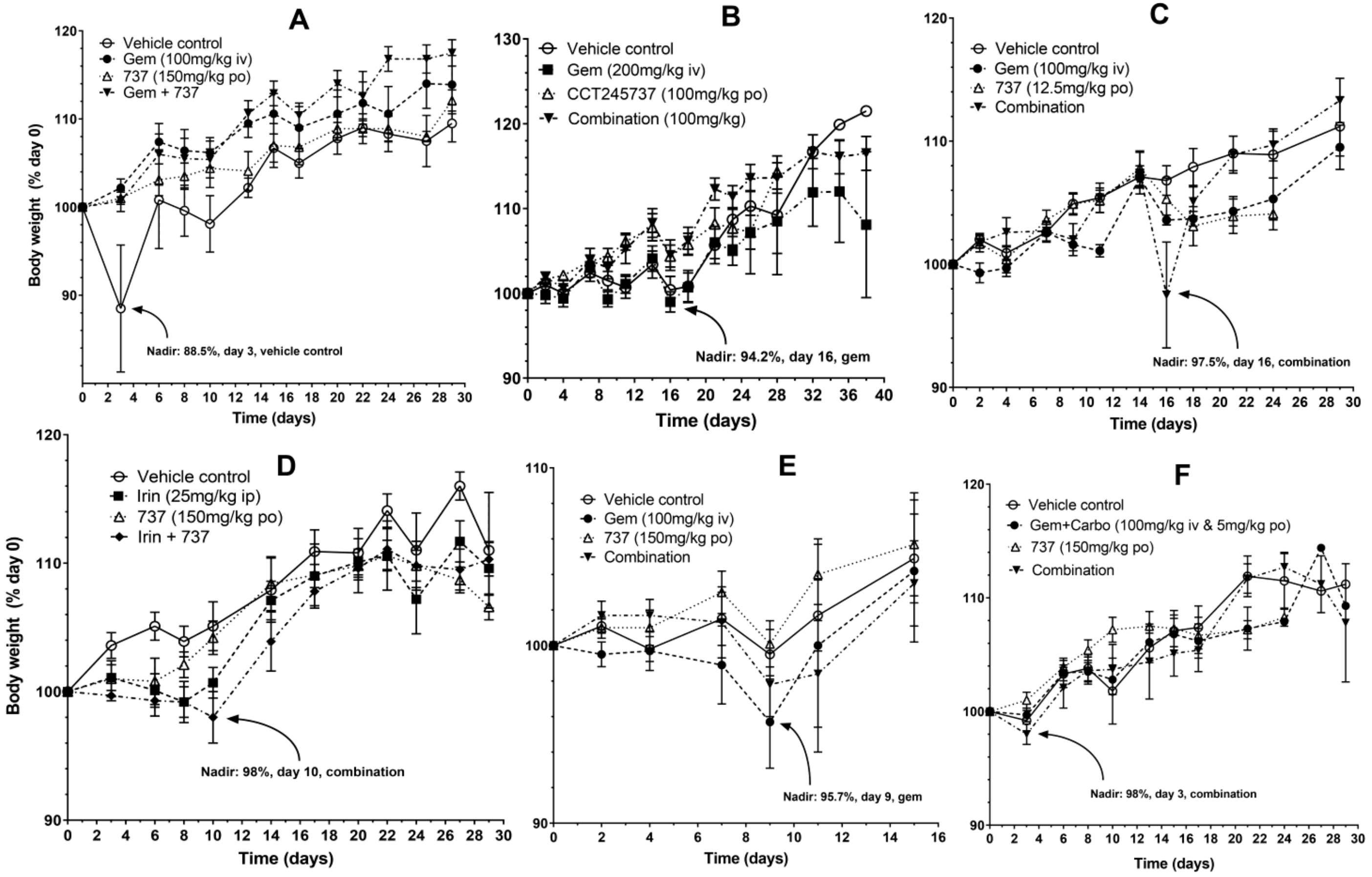

S Fig 5. Effect of CCT245737 and genotoxic drug treatment on animal body weights. A), B) and C) Relative body weights for mice bearing HT29 xenografts and treated with gemcitabine and CCT245737 (Expt 95, 62 and 11, respectively). D) As for A) but treated with Irinotecan and CCT245737 (Expt 96). E) Relative body weights for mice bearing SW620 xenografts and treated with gemcitabine and CCT245737 (Expt 94). D) Relative body weights for mice bearing Calu6 xenografts and treated with gemcitabine, carboplatin and CCT245737 (Expt 37). Values shown are mean±SE for 5-10 mice per point.
